# Supplementary material for: Estimating the burden of underdiagnosis within England: A modelling study of linked primary care data
Source: PLoS One. 2025 Jan 15;20(1):e0313877. doi: 10.1371/journal.pone.0313877 (PMC11734898; doi:10.1371/journal.pone.0313877)
Supplement: S1 Appendix — (DOCX) [file pone.0313877.s001.docx]

**S1 Appendix A. Defining case fatality rate for those undiagnosed**

We followed the approach below to define the case fatality rate among the undiagnosed. First, we define who is a disease case for a specific disease and who is not. Let us define who is a non-case first. For disease D, a non-case is someone with no risk of dying from disease D at a given time. Consequentially, a disease case is someone who has at least some risk of dying from disease D at a given point in time.

For the diseases we model, we assumed that the natural course of the disease without healthcare intervention is a progressive increase in the risk of dying from the disease over time (S1 Appendix A, Fig A1). Note that early in the course of disease D, this disease might not be diagnosable with current technology. During the course of the disease, there is a point in time and a level of excess mortality risk that the disease becomes diagnosable (Point A). This point is disease-specific; as technology improves, it moves to the left. Theoretically, we could use the risk of dying from disease D at Point A to define the “diagnosable” disease case and then find the probability of a “diagnosable” disease case being undiagnosed. The latter would be directly related to unmet need. Practically, Point A is very hard to observe even for diseases with mass screening programs because the likely treatment after diagnosis would modify the course of the disease.

However, to the right of this point, there is the point that the disease is diagnosed in real-world scenarios (Point B). We can approximate Point B by observing the disease-specific mortality of recently diagnosed individuals. In our study, we used the risk of dying from disease D at approximately Point B to define the “diagnosable” disease case and then find the probability of a “diagnosable” disease case being undiagnosed. Therefore, we used the case fatality rate among incident-diagnosed cases to define the case fatality rate among the undiagnosed.

Fig A1: Theoretical natural course of disease without intervention concerning disease-specific mortality over time
