# Supplementary material for: Estimating the burden of underdiagnosis within England: A modelling study of linked primary care data
Source: PLoS One. 2025 Jan 15;20(1):e0313877. doi: 10.1371/journal.pone.0313877 (PMC11734898; doi:10.1371/journal.pone.0313877)

**S2 Appendix B: Underdiagnosis by sex and disease, 2008 and 2018**

Fig B1: Underdiagnosis by sex and disease. England 2008 and 2018


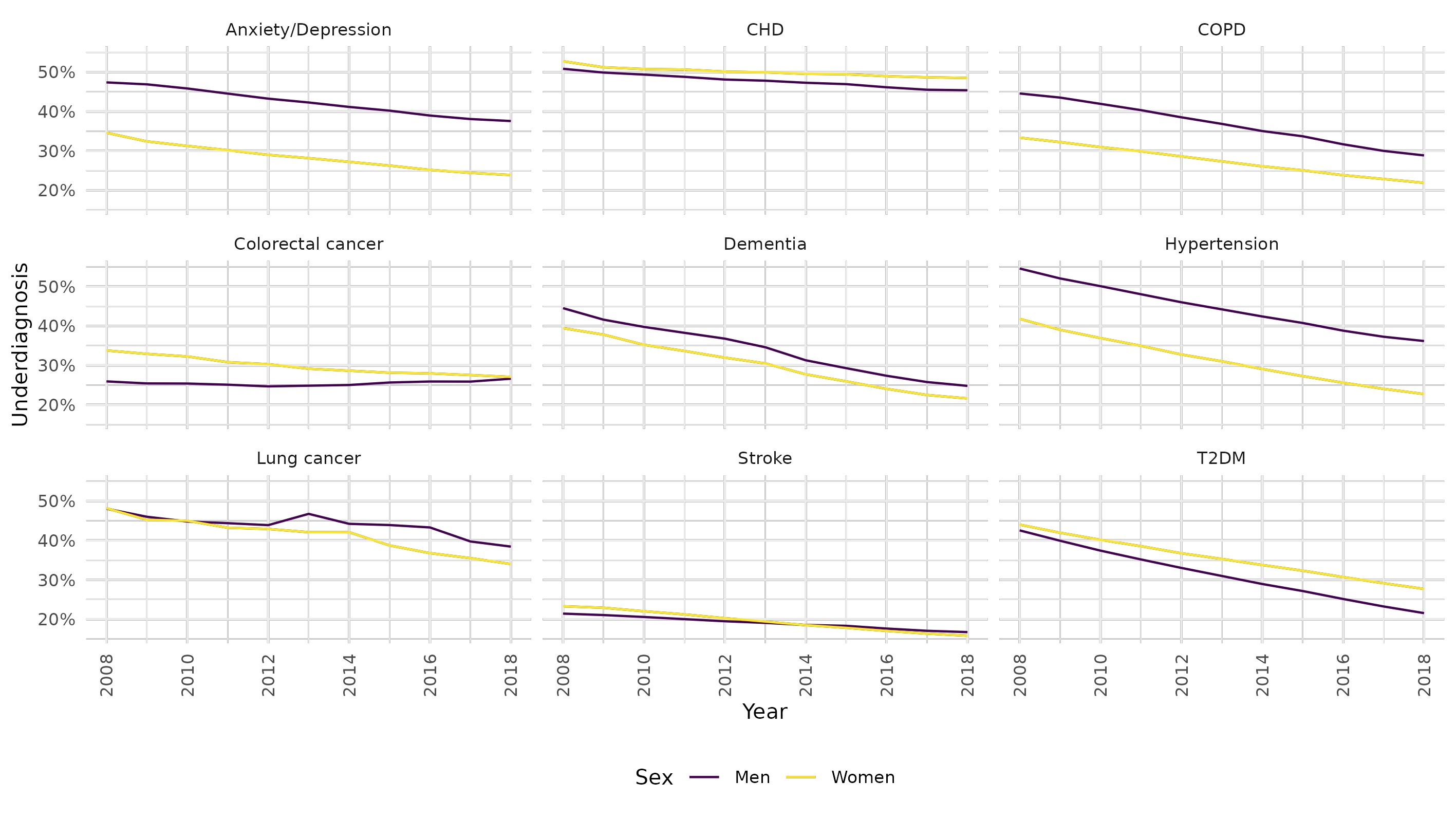

Supplement: S2 Appendix — (DOCX) [file pone.0313877.s002.docx]
