# Supplementary material for: Estimating the burden of underdiagnosis within England: A modelling study of linked primary care data
Source: PLoS One. 2025 Jan 15;20(1):e0313877. doi: 10.1371/journal.pone.0313877 (PMC11734898; doi:10.1371/journal.pone.0313877)
Supplement: S3 Appendix — (DOCX) [file pone.0313877.s003.docx]

**S3 Appendix C: Validation**

Fig C1 - T2DM validation: Underdiagnosis compared to HSE data, by Region and year


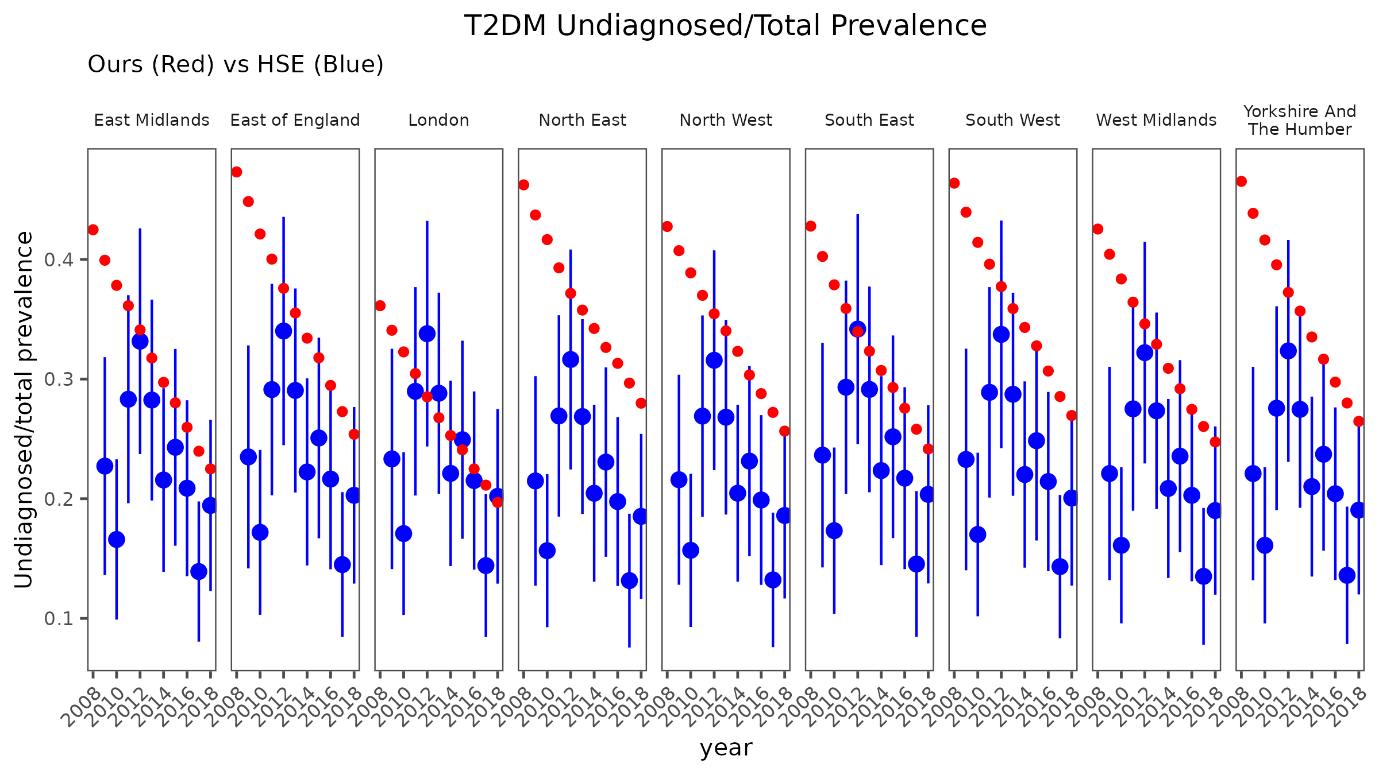


Fig C2 - T2DM validation: Underdiagnosis compared to HSE data, by IMD quintile


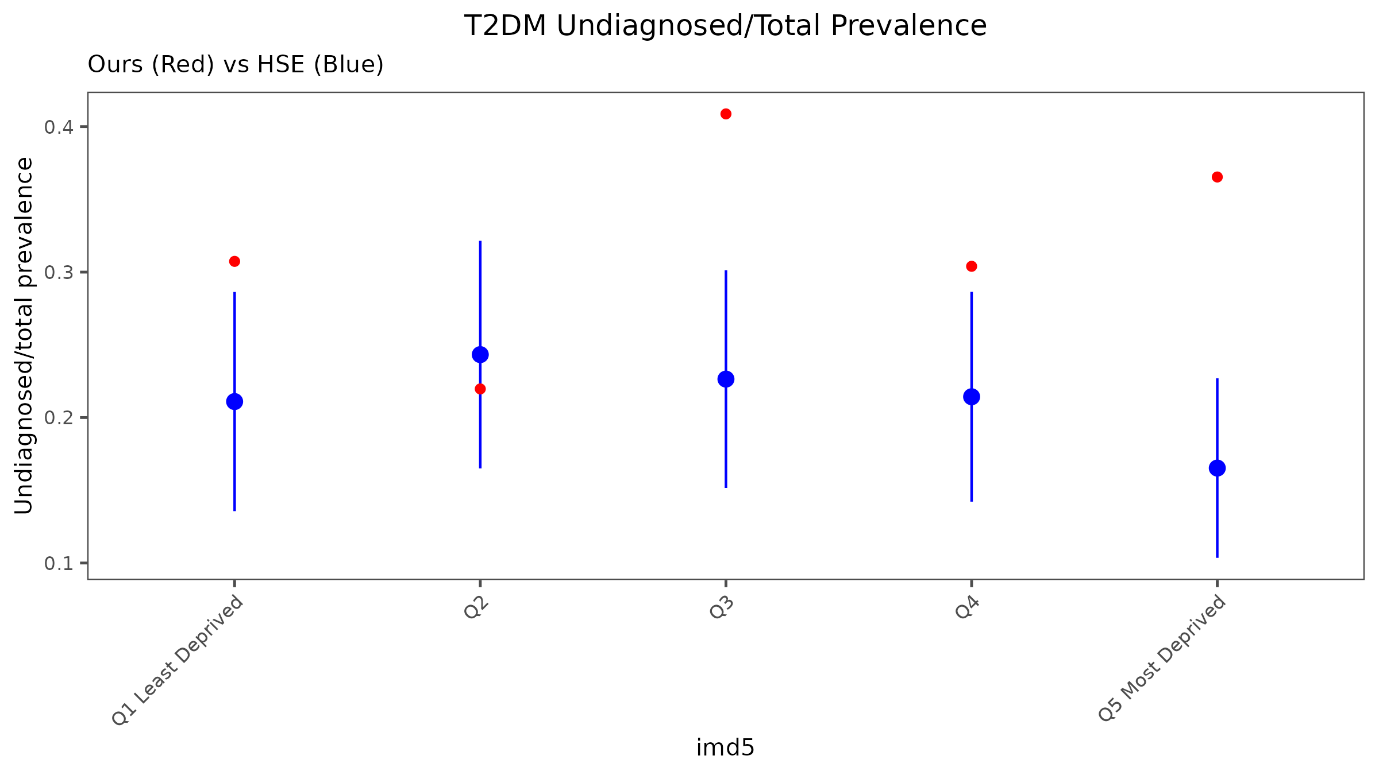


Fig C3 - T2DM validation: Underdiagnosis compared to HSE data, by age group


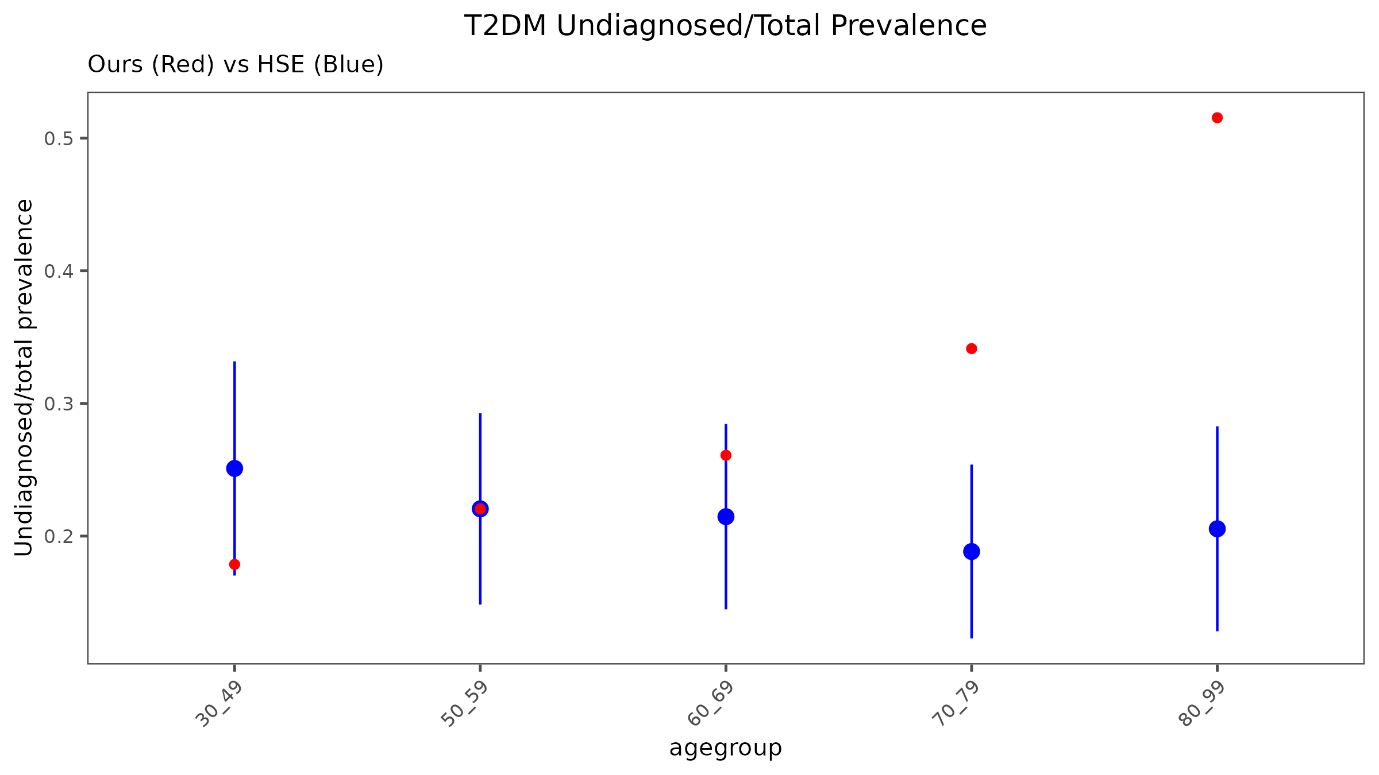


Fig C4 - Hypertension validation: Underdiagnosis compared to HSE data, by Region and year


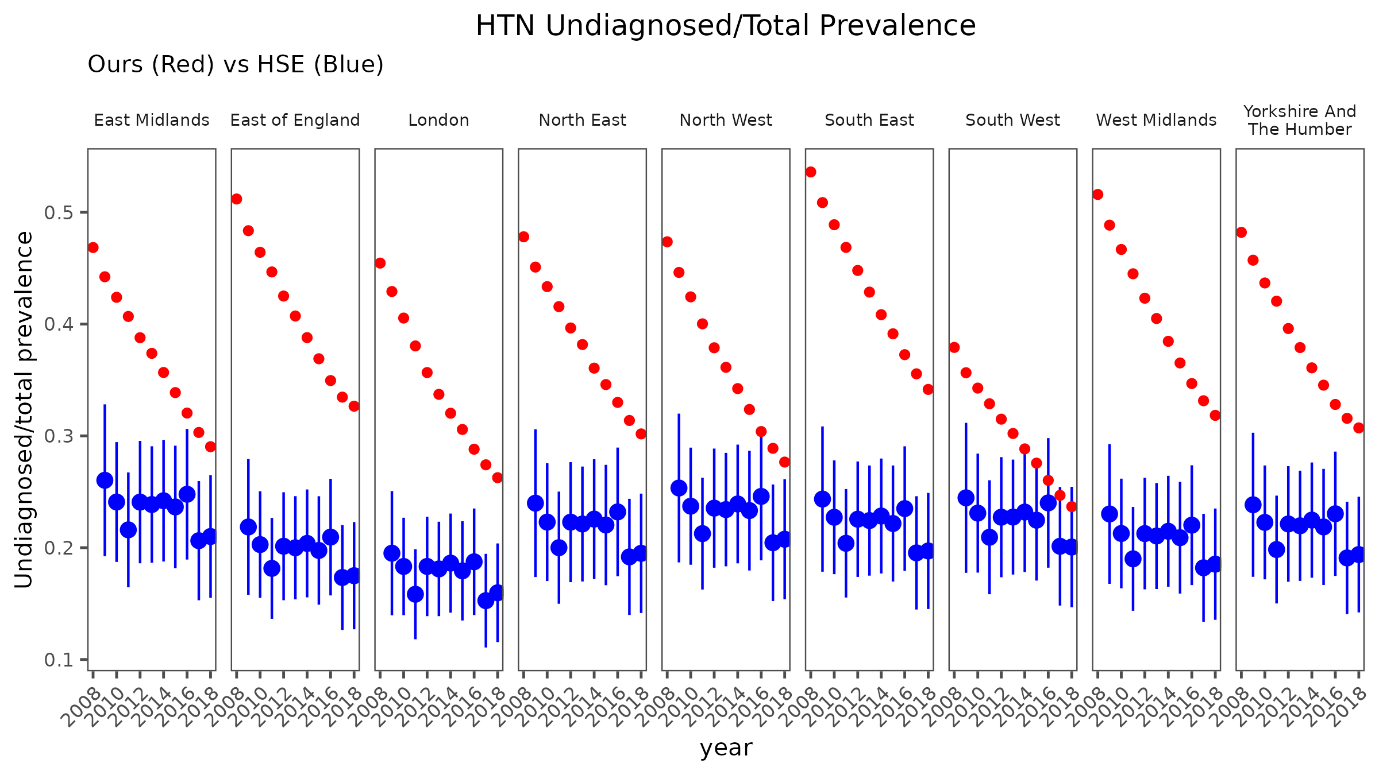


Fig C5 - Hypertension validation: Underdiagnosis compared to HSE data, by IMD quintile


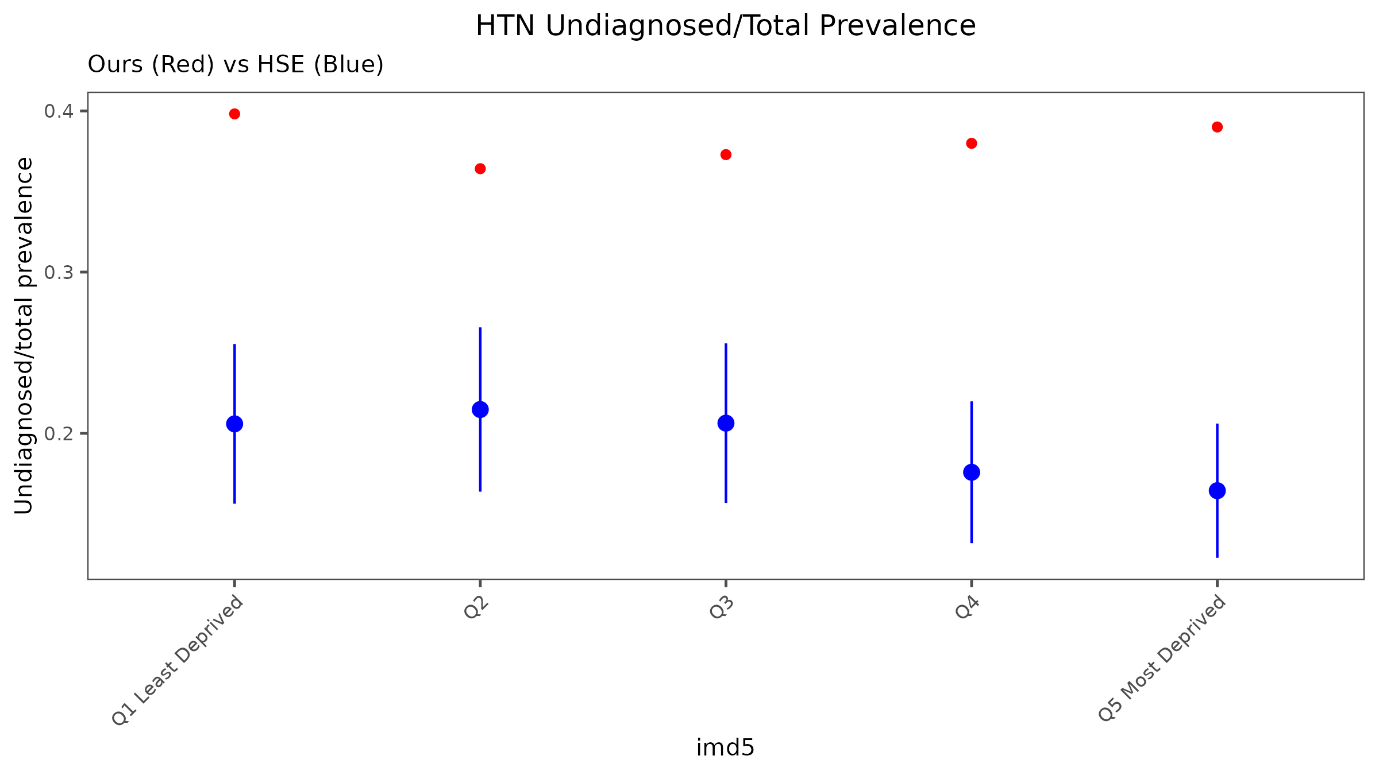


Fig C6 - Hypertension validation: Underdiagnosis compared to HSE data, by age group


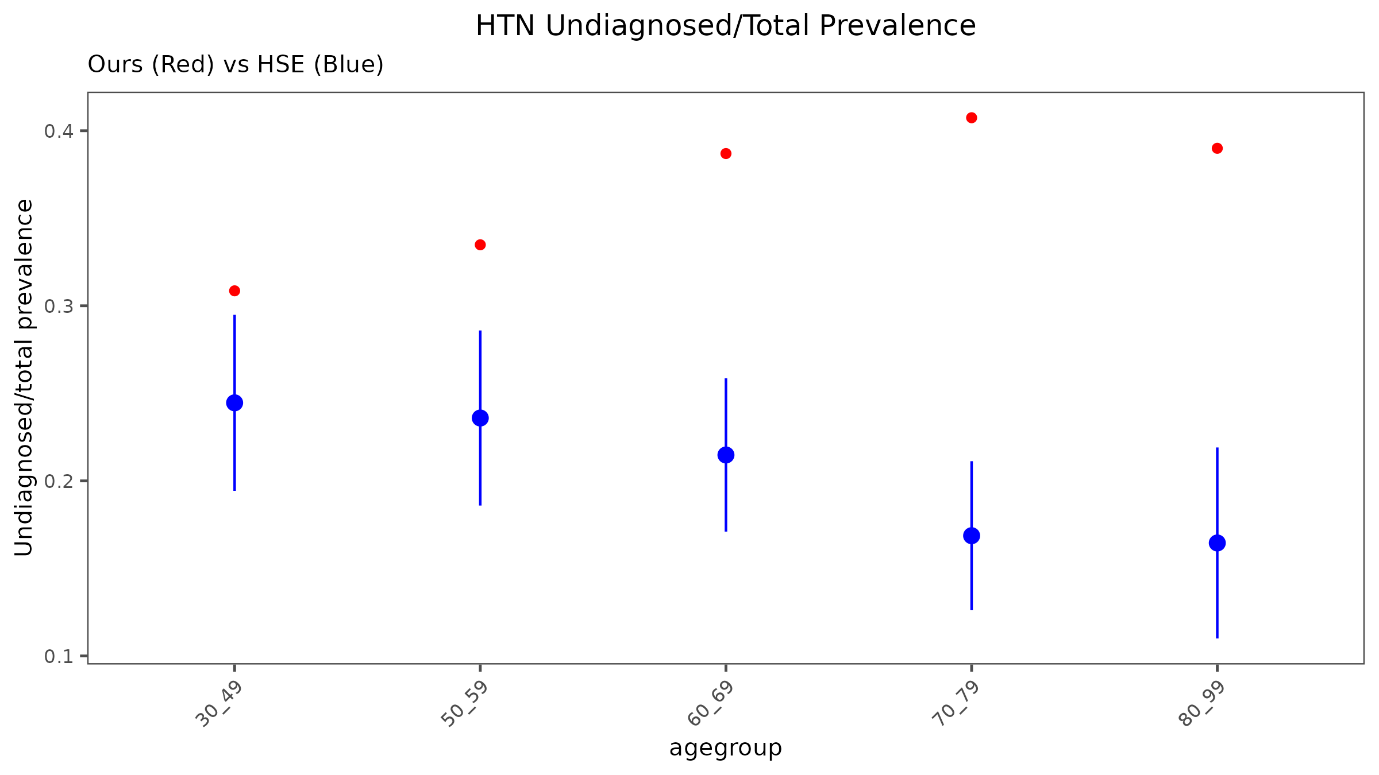


Fig C7 - Anxiety and depression validation: Underdiagnosis compared to Understanding Society data, by year and Region


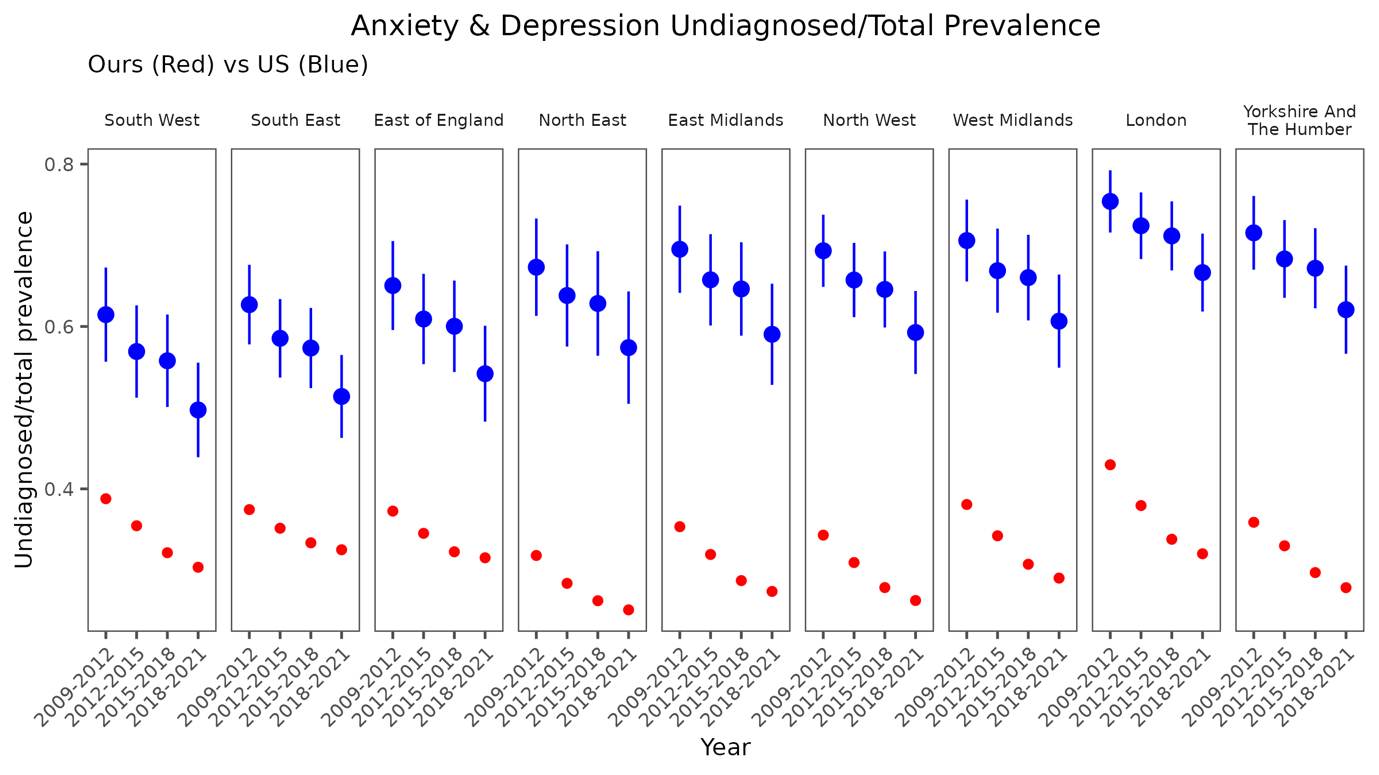


Fig C8 - Anxiety and depression validation: Underdiagnosis compared to Understanding Society data, by IMD quintile


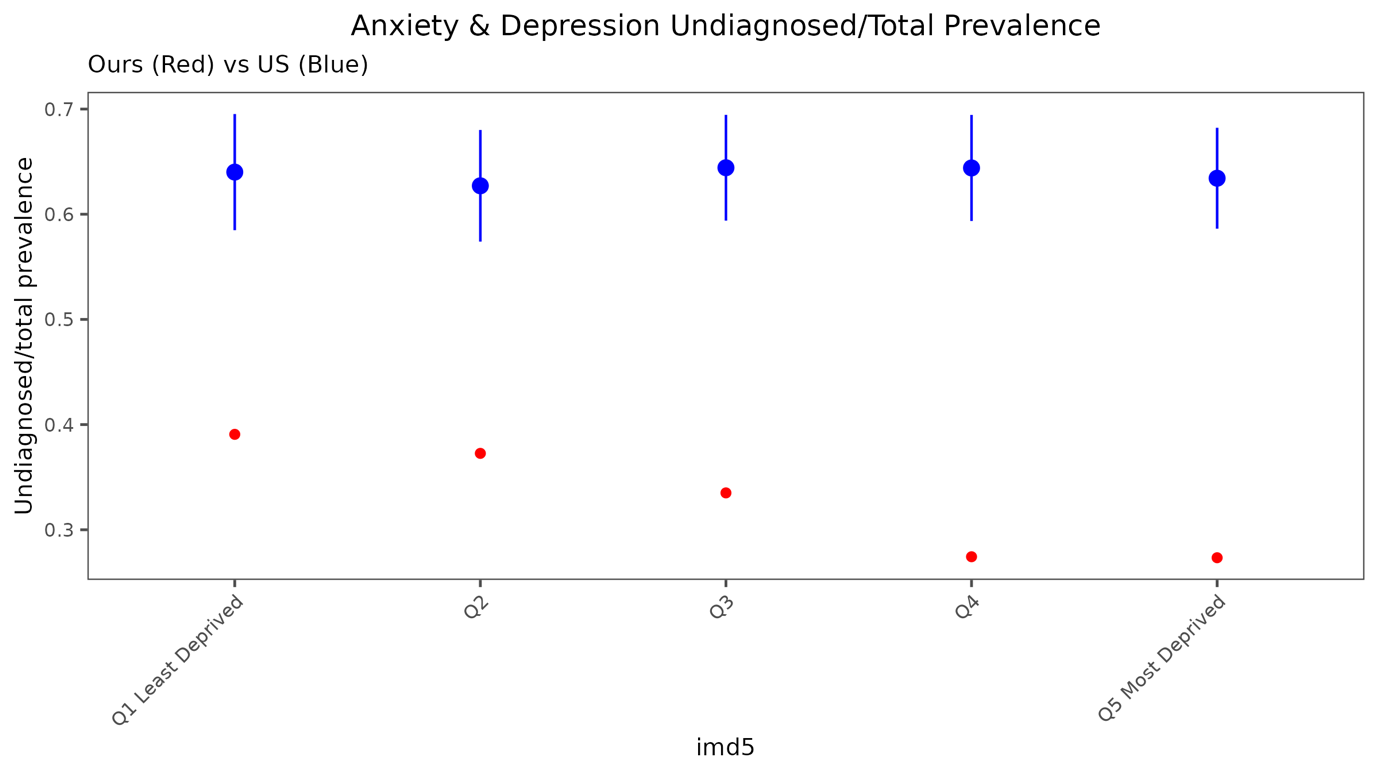


Fig C9 - Anxiety and depression validation: underdiagnosis compared to Understanding Society data, by age group


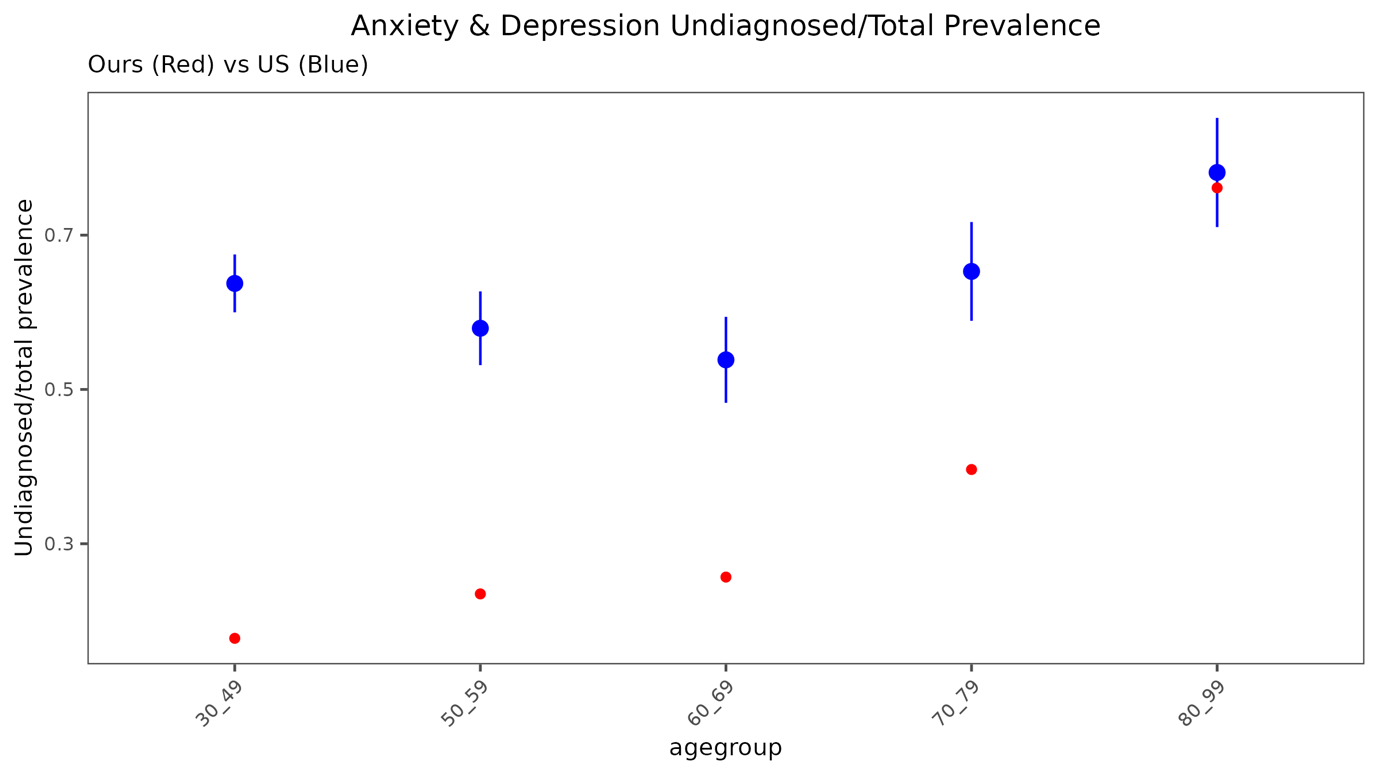


## Sensitivity analysis

### Comparing the distribution of the undiagnosed between CCGs under different modelling assumptions.

In our main model, we define individuals as dying of a disease if it is recorded as a primary or contributory cause on death certificates. This is likely to be a better reflection of overall morbidity, particularly where the primary cause of death may be cardiac or respiratory arrest or in older age groups, where multimorbidity may mean that multiple diseases contribute to death. To test whether using both primary and contributory causes of death, rather than just the primary cause, has a large effect on our estimated distribution of the undiagnosed, we compared estimates across CCGs using both approaches for each disease. The correlation coefficients between the two sets of estimates are given below in Table C1. For most diseases, this makes little difference, and there is a high level of correlation between the two sets of estimates. The exception could be breast cancer, where only considering the primary cause of death changed substantially the distribution of underdiagnosis between CCGs.

Table C1. The correlation between 2 sets of estimates of underdiagnosis from the epidemiological model (1) model using just the primary care of death and (2), model using primary and contributory causes of death (our main model).

| Disease | rho | p |
| --- | --- | --- |
| Anxiety/Depression | 0.890 | <0.001 |
| Breast cancer | 0.599 | <0.001 |
| CHD | 0.861 | <0.001 |
| COPD | 0.959 | <0.001 |
| Colorectal cancer | 0.924 | <0.001 |
| Dementia | 0.977 | <0.001 |
| Hypertension | 0.896 | <0.001 |
| Lung cancer | 1 | <0.001 |
| Prostate cancer | 0.998 | <0.001 |
| Stroke | 0.988 | <0.001 |
| T2DM | 0.909 | <0.001 |

In our main model, we estimated the number of undiagnosed individuals from the number of individuals in our data dying of the disease who were previously undiagnosed. To test whether our estimates are sensitive to using previously undiagnosed before death OR additionally include those diagnosed within the last year before death, we re-ran the model only using the previously undiagnosed before death, including those diagnosed within the last year before death. Table C2 compares estimates of the % undiagnosed across CCGs using both approaches for each disease, giving the correlation coefficients between the two sets of estimates. For most diseases, this makes little difference to the distribution of undiagnosed between CCGs, except for T2DM, where a relatively large number of cases of T2DM are only diagnosed in the year before death.

Table C2. The coefficients for correlations between 2 sets of estimates of underdiagnosis from the epidemiological model (1) model using just using previously undiagnosed before death (our main model) (2), model using both previously undiagnosed before death and those only diagnosed in the year before death.

| Disease | rho | p |
| --- | --- | --- |
| Anxiety/Depression | 0.994 | <0.001 |
| Breast cancer | 0.608 | <0.001 |
| CHD | 0.985 | <0.001 |
| COPD | 0.745 | <0.001 |
| Colorectal cancer | 0.892 | <0.001 |
| Dementia | 0.904 | <0.001 |
| Hypertension | 0.991 | <0.001 |
| Lung cancer | 0.873 | <0.001 |
| Prostate cancer | 0.977 | <0.001 |
| Stroke | 0.787 | <0.001 |
| T2DM | 0.389 | <0.001 |

C3 shows that alternative assumptions related to the case fatality rate in the undiagnosed, assuming it is equal to prevalent rather than incident diagnosed cases, do not have a large effect on the distribution of the undiagnosed between CCGs.

Table C3. The coefficients for correlations between 2 sets of estimates of underdiagnosis from the epidemiological model (1) model assuming the case fatality rate (CFR) is the same for the diagnosed and undiagnosed (our main model), and (2). Model assuming the CFR in the undiagnosed is the same as the CFR in people in the first year after diagnosis.

| Disease | rho | p |
| --- | --- | --- |
| Anxiety/Depression | 0.987 | <0.001 |
| Breast cancer | 0.992 | <0.001 |
| CHD | 0.925 | <0.001 |
| COPD | 0.971 | <0.001 |
| Colorectal cancer | 0.927 | <0.001 |
| Dementia | 0.738 | <0.001 |
| Hypertension | 0.989 | <0.001 |
| Lung cancer | 0.998 | <0.001 |
| Prostate cancer | 0.964 | <0.001 |
| Stroke | 0.997 | <0.001 |
| T2DM | 0.858 | <0.001 |
