# Supplementary material for: Estimating the burden of underdiagnosis within England: A modelling study of linked primary care data
Source: PLoS One. 2025 Jan 15;20(1):e0313877. doi: 10.1371/journal.pone.0313877 (PMC11734898; doi:10.1371/journal.pone.0313877)
Supplement: Attachment — Submitted filename: Response to Reviewers.docx [file pone.0313877.s004.docx]

Dear Editor and Reviewer,

Thank you for your invaluable and helpful comments and suggestions for this study. Based on your recommendations, we believe the revised version is greatly improved. Please find below our point-by-point response:

1. Please ensure that your manuscript meets PLOS ONE's style requirements, including those for file naming. The PLOS ONE style templates can be found at

https://journals.plos.org/plosone/s/file?id=wjVg/PLOSOne_formatting_sample_main_body.pdf and

<https://journals.plos.org/plosone/s/file?id=ba62/PLOSOne_formatting_sample_title_authors_affiliations.pdf>

Thank you. We made the necessary changes.

2. Please note that PLOS ONE has specific guidelines on code sharing for submissions in which author-generated code underpins the findings in the manuscript. In these cases, all author-generated code must be made available without restrictions upon publication of the work. Please review our guidelines at https://journals.plos.org/plosone/s/materials-and-software-sharing#loc-sharing-code and ensure that your code is shared in a way that follows best practice and facilitates reproducibility and reuse.

Thank you. We deposit our code at <https://github.com/ChristK/Unmet_need>.

3. When completing the data availability statement of the submission form, you indicated that you will make your data available on acceptance. We strongly recommend all authors decide on a data sharing plan before acceptance, as the process can be lengthy and hold up publication timelines. Please note that, though access restrictions are acceptable now, your entire data will need to be made freely accessible if your manuscript is accepted for publication. This policy applies to all data except where public deposition would breach compliance with the protocol approved by your research ethics board. If you are unable to adhere to our open data policy, please kindly revise your statement to explain your reasoning and we will seek the editor's input on an exemption. Please be assured that, once you have provided your new statement, the assessment of your exemption will not hold up the peer review process.

Thank you. We have updated the data statement to reflect that this study is based on third-party data that we cannot share. We now provide information on how researchers can access data from data providers. The modelling outputs that support the results in our paper are deposited on Zenodo with a provisional DOI: 10.5281/zenodo.14017677, and we will make them available publicly as soon as this paper gets accepted.

4. Please upload a new copy of Figure 4 as the detail is not clear. Please follow the link for more information: https://blogs.plos.org/plos/2019/06/looking-good-tips-for-creating-your-plos-figures-graphics/" <https://blogs.plos.org/plos/2019/06/looking-good-tips-for-creating-your-plos-figures-graphics/>"

Thank you. We have now fixed this technical glitch and reuploaded the figure.

5. Please review your reference list to ensure that it is complete and correct. If you have cited papers that have been retracted, please include the rationale for doing so in the manuscript text, or remove these references and replace them with relevant current references. Any changes to the reference list should be mentioned in the rebuttal letter that accompanies your revised manuscript. If you need to cite a retracted article, indicate the article’s retracted status in the References list and also include a citation and full reference for the retraction notice.

Thank you. We made the necessary changes in the formatting of the citations and references. We did not cite any retracted papers.

Reviewers' comments:

Reviewer #1: Dear Editor,

I read the manuscript titled “Estimating the burden of underdiagnosis within England: a modelling study of linked primary care data” with interest. As the authors very eloquently have laid out in the manuscript introduction the study is addressing an important unmet need for healthcare providers and policy makers. They have been very thorough in their modelling. The manuscript is methodologically sound and provides a novel way for exploring and understanding patterns of underdiagnosis in England. This research introduces a novel way of exploring this issue, it highlights the complexity of identifying drivers of underdiagnosis and has highlighted areas where further research is needed.

Thank you.

I only have some very minor comments for the authors.

Methods:

The ‘estimating underdiagnosis section’ could do with some reordering for flow. For example, in line 181 “except D as described above”, I think it should be as described below.

Thank you. Our statement is correct, and by ‘above’, we meant lines 165-169 but we now see how the wording was confusing. We have reworded this sentence for clarity, and it now reads, “All rates varied additionally by Region, except D, as we describe above regarding the imputation approach”.

The legend to Figure 1 should include definitions for A,B,C,D,E to aid the reader. Potentially consider defining these within the methods text as well.

Thank you. We have updated the legend to include A, B, C, D, and E definitions and also added the description in the text.

Discussion

The validation analyses suggest to me that the model overestimates the undiagnosed prevalence for most diseases (except for depression/anxiety). However the aim of the research was not to estimate the prevalence but rather to explore patterns. I think this should be explicit in the discussion. As it mitigates concerns around the magnitude of the problem and concentrates them on identified which populations / areas are most affected.

Thank you. We echo your suggestion, and we have now added to the first paragraph of the discussion the following text: “While our estimates of underdiagnosis appear higher than estimates from national surveys for T2DM and hypertension but lower than national survey estimates for anxiety and depression, the insights we get regarding patterns of underdiagnosis might still be useful and perhaps more relevant to policymaking than the absolute level of underdiagnosis. These patterns were consistent at large in our sensitivity analysis.”

Table 1. Please clarify that the proportions in the table are out of those that have the disease what proportion is undiagnosed.

Thank you. We updated the caption. It now reads “Underdiagnosis (probability of being undiagnosed given being diseased), for England 2008 and 2018.” We similarly updated all figure captions for consistency.

Figures:

Quality of Figure 4 needs improving as currently very pixelated.

Thank you. We have now fixed this technical glitch and reuploaded the figure.

All the best,

Chris Kypridemos

on behalf of the co-authors
